# *Brucella* Dysregulates Monocytes and Inhibits Macrophage Polarization through LC3-Dependent Autophagy

**DOI:** 10.3389/fimmu.2017.00691

**Published:** 2017-06-12

**Authors:** Yang Wang, Yuxiang Li, Haijun Li, Hongxiao Song, Naicui Zhai, Lixin Lou, Feng Wang, Kaiyu Zhang, Wanguo Bao, Xia Jin, Lishan Su, Zhengkun Tu

**Affiliations:** ^1^Institute of Translational Medicine, The First Hospital of Jilin University, Changchun, China; ^2^Department of Infectious Diseases, The First Hospital of Jilin University, Changchun, China; ^3^CAS Key Laboratory of Molecular Virology and Immunology, Institute Pasteur of Shanghai, Chinese Academy of Sciences, Shanghai, China; ^4^Lineberger Comprehensive Cancer Center, School of Medicine, University of North Carolina at Chapel Hill, Chapel Hill, NC, United States

**Keywords:** autophagy, brucellosis, inflammation, infection, cytokines

## Abstract

Brucellosis is caused by infection with *Brucella* species and exhibits diverse clinical manifestations in infected humans. Monocytes and macrophages are not only the first line of defense against *Brucella* infection but also a main reservoir for *Brucella*. In the present study, we examined the effects of *Brucella* infection on human peripheral monocytes and monocyte-derived polarized macrophages. We showed that *Brucella* infection led to an increase in the proportion of CD14^++^CD16^−^ monocytes and the expression of the autophagy-related protein LC3B, and the effects of *Brucella*-induced monocytes are inhibited after 6 weeks of antibiotic treatment. Additionally, the production of IL-1β, IL-6, IL-10, and TNF-α from monocytes in patients with brucellosis was suppressed through the LC3-dependent autophagy pathway during *Brucella* infection. Moreover, *Brucella* infection inhibited macrophage polarization. Consistently, the addition of 3-MA, an inhibitor of LC3-related autophagy, partially restored macrophage polarization. Intriguingly, we also found that the upregulation of LC3B expression by rapamycin and heat-killed *Brucella in vitro* inhibits M2 macrophage polarization, which can be reversed partially by 3-MA. Taken together, these findings reveal that *Brucella* dysregulates monocyte and macrophage polarization through LC3-dependent autophagy. Thus, targeting this pathway may lead to the development of new therapeutics against Brucellosis.

## Introduction

Brucellosis is a zoonotic infection caused by *Brucella*, a genus of Gram-negative bacteria that mostly infects animals including sheep, cattle, goats, pigs, and dogs. However, several *Brucella* species such as *Brucella abortus, Brucella melitensis, B. suis*, and *B. canis* can also infect humans (1). Brucellosis is one of the most common zoonotic bacterial infections worldwide, with over 500,000 new human cases annually and a prevalence rate exceeding 10/100,000 in some countries (2).

*Brucella* infection triggers a complex host response that involves both innate and adaptive immunity. More and more evidences show that host innate immune responses play critical roles in the control of *Brucella* infection (3). *Brucella* invades and replicates within monocytes and macrophages. To survive the hostile intracellular environment, *Brucella* uses several strategies (4). The lipopolysaccharide (LPS) within the *Brucella* cell outer membrane contains a special lipid A that assists the evasion from the host immune system during the early stages of infection (5). The toll-like receptors (TLRs) TLR-2, TLR-4, and TLR-9 are involved in the recognition of *Brucella* infection. TLR-2 is activated by lipidated outer membrane proteins (L-Omp16 and L-Omp19); TLR-4 is activated by *Brucella* unlipidated outer membrane proteins (U-Omp16) and LPS; and TLR-9 is activated by *Brucella* DNA (6). TLR activation leads to intracellular signaling *via* MyD88 and IRAK-4 resulting in the activation of NF-κB and MAPKs and consequent production of inflammatory cytokines (7). Cytosolic sensors such as NOD1 and NOD2 are also involved in the recognition of *Brucella* DNA (6).

After entering mammalian cells, *B. abortus* resides within a membrane-bound compartment, the *Brucella*-containing vacuole, which is covered from an endoplasmic reticulum derived replicative organelle under the control of the bacterium (8, 9). *Brucella* escapes from immune surveillance and establishes infection through (a) restriction of fusion between *Brucella*-containing vacuoles and lysosomal compartments to avoid degradation, (b) inhibition of apoptosis of infected mononuclear cells to extend its replication time, (c) prevention of dendritic cell maturation and thus the efficiency of antigen presentation, and (d) restriction of T cell activation (10). *Brucella* may also exploit the autophagic machinery to establish a favorable intracellular environment for its replication (8, 9).

Autophagy is a natural housekeeping mechanism through which superfluous or aged and damaged organelles are removed. It is also an important host defense mechanism that eliminates intracellular pathogens, as has been demonstrated for *Legionella pneumophila* and *Acinetobacter baumannii* (11, 12). However, some pathogens, such as *Mycobacterium tuberculosis* and *Listeria monocytogenes*, have evolved strategies to subvert autophagy for their own benefit (13, 14). The autophagy pathway can be a microorganism-friendly process that favors the intracellular survival of certain microorganisms. It exerts an anti-inflammatory effect through downregulation of the inflammasome, which promotes the maturation of pro-inflammatory cytokines interleukin IL-1β and IL-18 (15, 16).

Monocytes/macrophages are part of the innate immune system that provides the first line of defense against pathogens. Through phagocytosis and antigen presentation, they play important roles in immune surveillance and immune regulation (17). Peripheral monocytes tend to polarize to different subtypes of macrophages according to the tissue microenvironment. The Th1 cytokine IFN-γ and TLR-4 agonist LPS polarize monocytes to the classical activated macrophages (M1 macrophages), which produce pro-inflammatory cytokines such as TNF-α and IL-12. M1 macrophages clear pathogens but also cause tissue damage. In contrast, upon exposure to Th2 cytokines such as IL-4, monocytes differentiate into alternatively activated macrophages (M2 macrophages) that produce anti-inflammatory mediators such as IL-10. M2 macrophages suppress inflammation and facilitate wound healing (18). The extent of tissue inflammation developed as a consequence of an innate immune response is determined in large part by the balance between the pro-inflammatory M1 and anti-inflammatory M2 macrophages (19). It was reported that *Brucella* infection prevents the apoptosis of monocyte and macrophage and modulates macrophage functions (20–23). However, the effect of *Brucella* infection on macrophage polarization remains to be elucidated.

In this study, we investigated the effect of *Brucella* on peripheral monocytes and the polarization of macrophages. Our results indicated that infection with *Brucella* leads to an increase in CD14^++^CD16^−^ monocytes and a decrease in CD14^+^CD16^+^ monocytes, as well as an increase in the expression of the autophagy-related protein LC3B in all monocyte subsets obtained from brucellosis patients. *Brucella* dysregulates monocytes and inhibits macrophage polarization through an upregulation of LC3-dependent autophagy process.

## Materials and Methods

### Human Blood Samples

Diagnoses of acute brucellosis were made based on history, clinical presentation, and laboratory tests (1). Patients who had contact history with cattle, sheep, or deer and clinical manifestation of intermittent fever with or without arthralgia during the last 3 months were subjected to laboratory testing. Rose bengal test positive, serum agglutination titer >1:160, and PCR test [Diagnostic Kit for Brucella DNA Kit (Jiangsu Bioperfectus Technologies, Taizhou, China)] positive were confirmation of brucellosis. All the patients were also examined by chest tomography and tuberculosis T-SPOT test to exclude tuberculosis. Twenty-five patients diagnosed with acute brucellosis and 15 healthy individuals were included in this study (Table 1). All patients were treated with rifampicin and doxycycline for 6 weeks, and five of them were recalled for posttreatment follow-up and collection of blood samples. The demographic data and clinical characteristics before and after treatment are shown in Table 2. The venous blood from patients and healthy controls (HC) in terms of diagnosis were collected before and after treatment. The study was approved by the Human Research Ethics Committee of the First Hospital of Jilin University, Jilin Province, China. All adult subjects provided written informed consent, and no children were included in the study.

**Table 1:** Demographics and clinical characteristics of patients with brucellosis and healthy controls.

	Brucellosis patients	Health controls	*t*-test
*n*	25	15	
Male/female	15/10	8/7	
Age (mean ± SD)	42.9 ± 18.1	31.8 ± 8.66	ns
Fever	98%	0%	
Arthralgia	50%	0%	
CNS	4%	0%	
CRP (mean ± SD)	45.53 ± 22.14	1.93 ± 0.2815	*P* < 0.001
ESR (mean ± SD)	40.65 ± 17.45	7.75 ± 0.9435	*P* < 0.001
AST (mean ± SD)	38.94 ± 22.80	20.6 ± 1.545	*P* < 0.001
ALT (mean ± SD)	37.80 ± 23.01	16.7 ± 1.328	*P* < 0.001
Positive rose bengal agglutin	100%	0%	
Positive serum agglutination	100%	0%	
PCR test	100%	0%	

*CNS, central nervous system; CRP, C-reactive protein; ESR, erythrocyte sedimentation rate; AST, aspartate aminotransferase; ALT, alanine aminotransferase; PCR, polymerase chain reaction. P values of <0.05 were considered statistically significant. ns, not significant*.

**Table 2:** The demographics and clinical characteristics of patients with brucellosis before and after treatment.

	Before treatment	After treatment	*t*-test
*n*	5		
Male/female	3/2		
Age (mean ± SD)	49 ± 11.34		
Fever	100%	0%	
Arthralgia	40%	0%	
CRP (mean ± SD)	39.84 ± 17.24	3.86 ± 1.179	*P* < 0.01
ESR (mean ± SD)	35 ± 10.63	11.8 ± 2.538	*P* < 0.01
AST (mean ± SD)	33.80 ± 6.22	23.8 ± 3.68	ns
ALT (mean ± SD)	29.4 ± 2.19	21.6 ± 2.40	ns
PCR test	100%	0%	

*CNS, central nervous system; CRP, C-reactive protein; ESR, erythrocyte sedimentation rate; AST, aspartate aminotransferase; ALT, alanine aminotransferase; PCR, polymerase chain reaction. P values of <0.05 were considered statistically significant. ns, not significant*.

### The Preparation of Heat-Killed *Brucella* (HK-Br)

The smooth strain *B. abortus* 2308 was grown overnight in tryptic soy broth, harvested by centrifugation, and washed twice in phosphate-buffered saline (PBS). Bacterial numbers in cultures were estimated by comparing the OD at 600 nm with a standard curve, but the actual concentration of inocula was checked by plating on tryptic soy agar (TSA) plates. All live *Brucella* manipulations were performed in biosafety level 3 facilities (China CDC, Beijing). To prepare HK-Br, bacteria were washed in sterile PBS, heat killed at 70°C for 30 min, aliquoted, and stored at −70°C until used. The absence of *B. abortus* viability after heat killing was verified by the absence of bacterial growth on TSA.

### Cell Isolation and Purification

PBMCs were isolated by density centrifugation using Lymphoprep (Fresenius Kabi Norge AS, Halden, Norway) as previously described (24). Monocytes were purified from PBMCs using a Human CD14 MicroBead Kit (Miltenyi Biotec, Bergisch Gladbach, Germany) as previously described (25). The purity of the CD14^+^ cells was >95% as determined by flow cytometry.

### Cell Culture

Purified monocytes (2 × 10^6^/ml) were stimulated with 1.0 µg/ml LPS from *E. coli* O111:B4 (Sigma-Aldrich Inc., St. Louis, MO, USA) as previously described (26, 27). Monocytes were differentiated to M1/M2 macrophages as previously described with modification (25). Briefly, purified monocytes (2 × 10^6^/ml) were cultured with GM-CSF (400IU/ml) or M-CSF (50 ng/ml) for 5 days in RPMI 1640 medium (Invitrogen, Carlsbad, CA, USA) supplemented with 10% heat-inactivated fetal calf serum, 100 IU/ml penicillin and 100 µg/ml streptomycin. For M1 polarization, GM-CSF-induced macrophages were cultured with LPS (100 ng/ml) and IFN-γ (20 ng/ml) for an additional 24 h. For M2 polarization, M-CSF-induced macrophages were further cultured with IL-4 (25 ng/ml) and IL-13 (25 ng/ml) for an additional 24 h. All the recombination human cytokines were purchased from R&D (Minneapolis, MN, USA).

To investigate the involvement of autophagy in monocytes and macrophage polarization, purified monocytes were pretreated with an autophagy inhibitor, 3 µM 3-MA (Sigma-Aldrich, St. Louis, MO, USA) for 3 h, and then cultured for 24 h with HK-Br (MOI = 100:1), 100 nM rapamycin (Sigma-Aldrich, St. Louis, MO, USA) was used as a positive control for autophagy as previous described (12).

### Flow Cytometry

Cell staining and flow cytometry analysis were performed as described (25). Briefly, differentiated M1 and M2 macrophages were characterized by staining with the following antibodies: mouse anti-human CD14-FITC, mouse anti-human CD16-PE-Cy7, mouse anti-human CD80-PE, mouse anti-Human CD86-FITC, mouse anti-human CD163-PE, and mouse anti-Human CD206-FITC. Markers of monocytes were mouse anti-human CD282-FITC, CD283-PE, and mouse anti-human CD284-APC. Mouse anti-human LC3B-PE was used as a marker for the autophagy-related protein (28). Intracellular staining was performed using the following mouse anti-human mAb: TNF-α, IL-6, IL-1β, and IL-10. All the antibodies were purchased from BD Biosciences (San Jose, CA, USA), except for anti-LC3B antibody (Cell Signaling Technology, Danvers, MA, USA), and used at a dilution of 1:50. Flow cytometry was performed using a BD Biosciences FACS CANTOII flow cytometer (BD Biosciences). The data acquired were analyzed with FlowJo (Treestar software, Ashland, OR, USA).

### Western Blotting

Western blotting was performed according to the published method (9, 12). Samples (50 µg total protein/lane) were loaded onto SDS-PAGE gels in this experiment. The primary (rabbit anti-human LC3, rabbit anti-human Beclin-1 and rabbit anti-human β-actin antibody) and secondary (anti-rabbit horseradish peroxidase-labeled antibody) antibodies were purchased from Cell Signaling Technology and used at a dilution of 1:1,000 and 1:2,000, respectively. The images were obtained using a CanoScan LiDE 100 scanner (Canon). The results were quantified using Image-J software.

### Enzyme-Linked Immunosorbent Assay (ELISA)

Enzyme-linked immunosorbent assay was performed with human IL-1β, IL-6, IL-10, and TNF-α ELISA Ready-SET-Go Kits (eBioscience, San Diego, CA, USA) according to the manufacturer’s instructions.

### Statistical Methods

All data were analyzed using the D’Agostino and Pearson omnibus normality test. *P* values of <0.05 were considered statistically significant. Mean values were compared using either a paired *t*-test (two groups) or ANOVA (more than two groups), followed by a Bonferroni correction for multiple comparisons test. All statistical tests were performed using GraphPad Prism software (San Diego, CA, USA).

## Results

### *Brucella* Infection Increased the Proportion of CD14^++^CD16^−^ Monocytes

**Figure 1:**
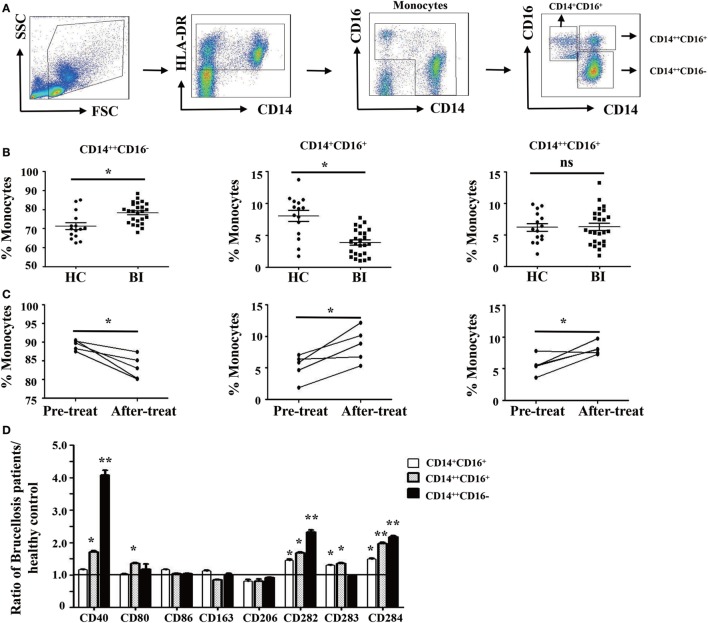
Characteristics of monocyte subsets in patients with brucellosis and healthy control subjects. **(A)** Gating strategy for monocyte subsets. **(B)** The percentage of each monocyte subset in patients with *Brucellia* infection (BI) (*n* = 25) and healthy controls (HC) (*n* = 15). **(C)** The percentage of monocyte subsets in five brucellosis patients before and after a 6-week treatment with rifampicin and doxycycline. **(D)** The phenotypic comparisons of monocyte subsets were normalized as the ratio of brucellosis patients/HC. *P < 0.05. **P < 0.01; **P < 0.001.

*Brucella* establishes infection in host monocytes and macrophages. To determine whether *Brucella* infection affects the frequency and phenotype of monocyte subsets, the monocytes were divided into three subsets based on CD14 and CD16 expression (29) as gating strategy shown in Figure 1A. We observed that the proportion of CD14^++^CD16^−^ monocytes in patients with *Brucella* infection was significantly higher than in HC, whereas the proportion of CD14^+^CD16^+^ monocytes was significantly lower. There was no significant difference in the CD14^++^CD16^+^ monocyte subset between the two groups (Figure 1B).

We further compared the frequency and phenotype of monocyte subsets in five patients with brucellosis before and after treatment with rifampicin and doxycycline. Results showed that the proportion of CD14^++^CD16^−^ monocytes significantly decreased after 6 weeks of antibiotic treatment, whereas both CD14^+^CD16^+^ and CD14^++^CD16^+^ monocytes increased significantly (Figure 1C).

To further characterize the phenotypic difference of monocyte subsets between patients with *Brucella* infection and healthy control individuals, cell surface expression of antigen presenting markers: CD1a and CD1b, costimulatory molecules: CD40, CD80, and CD86, M2 macrophage markers: CD163 and CD206, and TLR: CD282 (TLR-2), CD283 (TLR-3), and CD284 (TLR-4) were examined by flow cytometry. Results in Figure 1D showed that the expression levels of CD40, CD282, and CD284 on all three monocyte subsets are statistically higher in patients with *Brucella* infection than in healthy control individuals, whereas no significant differences were found in CD1a, CD1b, CD80, CD86, CD163, CD206, and CD283 expression levels for the various monocyte subsets between patients and controls.

### *Brucella*-Induced LC3-Dependent Autophagy in Monocytes

**Figure 2:**
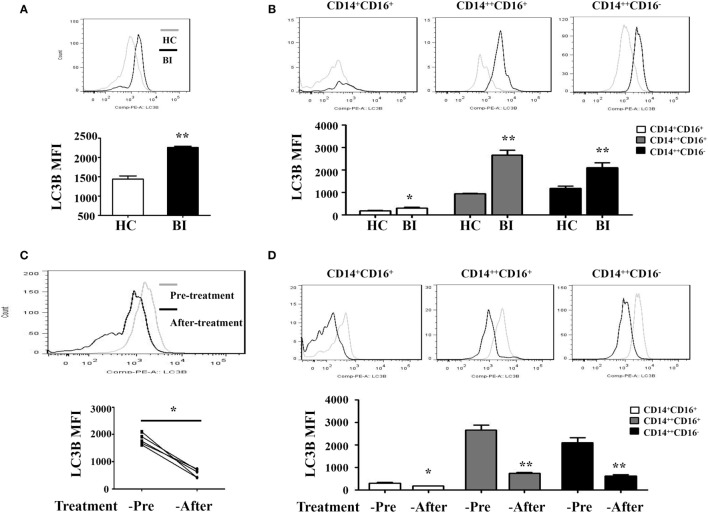
LC3B expression on monocytes of patients with brucellosis. LC3B expression levels on monocytes were analyzed by flow cytometry. LC3B expression levels detected in PBMCs by flow cytometry in 25 brucellosis patients and 15 healthy volunteers were included. The results were presented as the mean fluorescence intensity. **(A)** LC3B expression levels on monocytes from patients with *Brucellia* infection (BI) (*n* = 25) and healthy controls (HC) (*n* = 15). **(B)** LC3B expression levels on different monocyte subsets. **(C,D)** LC3B expression levels in monocytes were detected by flow cytometry using PBMCs from five patients before and after a 6-week treatment with rifampicin and doxycycline. **(C)** LC3B expression levels of monocytes from brucellosis patients (*n* = 5). **(D)** LC3B expression levels on different monocyte subsets. **P < 0.01; **P < 0.001.

Recent studies have shown that many human diseases result from overzealous innate immune responses including autophagy (30). Therefore, we investigated whether *Brucella* infection induces monocyte autophagy by quantify the expression of an autophagy-related protein LC3B using flow cytometry in patients with *Brucella* infection and in healthy control individuals. Results shown in Figure 2A demonstrated a higher LC3B expression level on the entire monocyte population from patients with *Brucella* infection than that from healthy control individuals, as well as monocyte subsets that are either CD14^++^CD16^+^ or CD14^++^CD16^−^ (Figure 2B). It was reported that rifampicin inhibits rapamycin-induced autophagy (31). We further examined whether anti-*Brucella* treatment could modulate LC3B expression on monocytes. Figure 2C showed a significant downregulation of LC3B expression levels on the overall monocyte population after 6 weeks of treatment of brucellosis with rifampicin and doxycycline (*P* < 0.001), and the same is true for each of the three monocyte subsets (Figure 2D).

**Figure 3:**
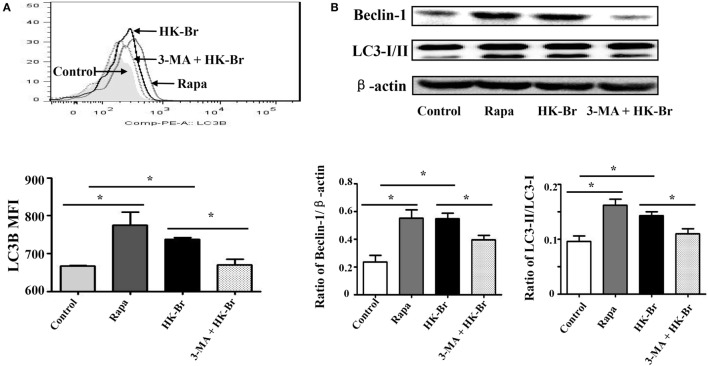
Heat-killed *Brucella* (HK-Br) induced LC3-dependent autophagy. **(A)** Purified monocytes from five healthy volunteers were pretreated with rapamycin (100 nM, 12 h) or HK-Br (MOI = 100:1) for 24 h with or without 3-MA pretreatment (3 mM, 3 h), and the LC3B levels were detected by flow cytometry. **(B)** Western blotting of LC3 and Beclin-1 in pretreated cells were performed, and the ratios of LC3-II/LC3-I and Beclin-1/β-actin were calculated. *P < 0.05; **P < 0.01.

To determine whether *Brucella* infection induces LC3-dependent autophagy in monocytes, purified monocytes from healthy donors were cultured for 24 h with HK-Br (a possible autophagy inducer) in the presence or absence of 3-MA, and rapamycin was used as a positive control for autophagy (12). LC3B expression levels of monocytes were determined by flow cytometry. The results showed that both HK-Br and rapamycin significantly induced LC3B expression of monocytes compared with untreated monocytes, whereas 3-MA significantly inhibited HK-Br-induced LC3B expression (Figure 3A). Beclin-1 was regarded as an important marker of autophagy as LC3 (12). Most autophagic responses were Beclin-1 dependent. The expression levels of LC3-I/II and Beclin-1 were also examined by western blotting, and β-actin was used as internal reference protein. The ratio of LC3-II/LC3-1 and Beclin-1/β-actin were calculated. Consistently, both HK-Br and rapamycin increased the ratios of LC3-II/LC3-I and Beclin-1/β-actin, and 3-MA decreased these ratios (Figure 3B). Collectively, these results indicate that *Brucella* infection induces autophagy-related protein LC3B expression in monocytes.

### *Brucella* Infection Inhibits the Function of Monocytes *via* Autophagy

**Figure 4:**
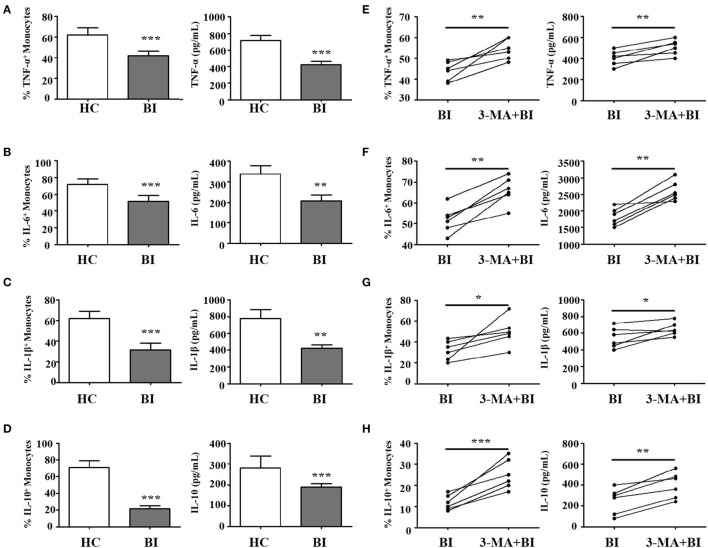
*Brucella* infection inhibited the function of monocytes *via* autophagy. Purified monocytes from 10 patients with *Brucellia* infection (BI) and 10 healthy controls (HC) were stimulated with lipopolysaccharide (LPS) (1 µg/ml, 6 h). **(A)** TNF-α, **(C)** IL-6, **(E)** IL-1β, and **(G)** IL-10 expression levels on monocytes and their production by monocytes were examined by intracellular cytokines staining and enzyme-linked immunosorbent assay (ELISA), respectively. Purified monocytes from six brucellosis patients were pretreated with 3-MA (3 mM, 3 h), and then stimulated with LPS. **(B)** TNF-α, **(D)** IL-6, **(F)** IL-1β, and **(H)** IL-10 expression levels on monocytes and their production by monocytes were examined by intracellular cytokines staining and ELISA, respectively. *P < 0.05, **P < 0.01, ***P < 0.001.

In response to pathogens, monocytes produce pro-inflammatory cytokines including IL-1β, IL-6, IL-8, and TNF-α, and anti-inflammatory cytokine such as IL-10 and TGF-β. To determine whether *Brucella* infection regulates monocyte function, purified monocytes from healthy donors and brucellosis patients were stimulated by LPS, with or without pretreatment with 3-MA. The expression and secretion of pro-inflammatory (IL-1β, IL-6, TNF-α) and anti-inflammatory (IL-10) cytokines were then examined by flow cytometry and by ELISA. Results in Figure 4 showed that upon LPS stimulation, monocytes from brucellosis patients expressed lower levels of TNF-α (Figure 4A), IL-6 (Figure 4B), IL-1β (Figure 4C), and IL-10 (Figure 4D) than those from HC. Conversely, 3-MA treatment that inhibited autophagy led to elevated expression of TNF-α (Figure 4E), IL-6 (Figure 4F), IL-1β (Figure 4G), and IL-10 (Figure 4H) than those from untreated monocytes from brucellosis patients. These results indicated that cytokine expression and production from monocytes is impaired after *Brucella* infection, but partially restored by the autophagy inhibitor.

### *Brucella* Infection Inhibits Macrophage Polarization *via* Autophagy

**Figure 5:**
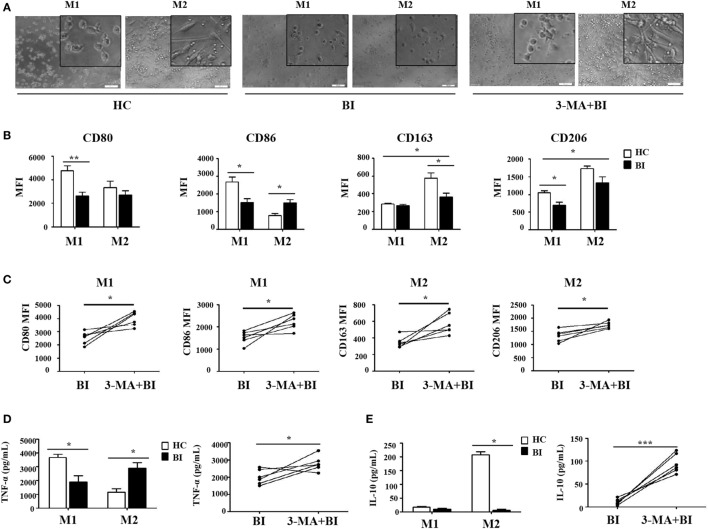
*Brucella* infection inhibited macrophage polarization *via* autophagy. Monocytes were purified from the peripheral blood of 10 patients with *Brucellia* infection (BI) and 10 healthy controls (HC), and purified monocytes from 6 patients with BI were also pretreated with 3-MA. These monocytes were polarized to M1 and M2 macrophages. **(A)** Morphology was assessed by microscopy (scale bar 20 µm). **(B)** CD80, CD86, CD163, and CD206 expression of M1 and M2 macrophages from brucellosis patients and healthy volunteers were analyzed by flow cytometry. **(C)** CD80 and CD86 expression of M1 macrophages, CD163 and CD206 expression of M2 macrophages were determined by flow cytometry. **(D)** TNF-α and **(E)** IL-10 production were detected by enzyme-linked immunosorbent assay. *P < 0.05, **P < 0.01, ***P < 0.001.

To further test whether *Brucella* infection alters monocyte polarization to M1/M2 macrophages, isolated monocytes were polarized to M1 and M2 macrophages in the presence or absence of 3-MA, and analyzed by flow cytometry. Results in Figure 5 showed that M1 and M2 macrophages polarization was phenotypically different between monocytes obtained from patients with *Brucella* and those from healthy individuals. As expected, the polarization to M1/M2 macrophages in the presence of 3-MA was similar irrespective of the source of monocytes. Morphologically, M1 macrophages polarized from healthy individuals had a round-shaped appearance, while M2 macrophages exhibited a long spindle shape. In contrast, M1 macrophages polarized from patients with *Brucella* infection had a smaller round-shaped appearance, and M2 macrophages lacked the long spindle shape (Figure 5A). CD80 and CD86 were highly expressed on M1 macrophages, while CD163 and CD206 were highly expressed on M2 macrophages in healthy individuals. However, there were no obvious differences in CD80 and CD86 expression levels between M1 and M2 macrophages in patients with *Brucella* infection. Additionally, CD80 and CD86 expression levels of M1 macrophages in patients with *Brucella* infection were significantly lower than those of healthy individuals (Figure 5B). In addition, the expression levels CD163 and CD206 on M2 macrophages were higher than on M1 macrophages in patients with *Brucella* infection (Figure 5B). CD163 expression level on M2 macrophages in patients with *Brucella* infection was significantly lower than those of healthy control individuals, but CD206 expression level on M2 macrophages exhibited no such a difference. Moreover, 3-MA restored CD80 and CD86 expression levels on M1 macrophages, and CD163 and CD206 expression levels on M2 macrophages in brucellosis patients (Figure 5C).

The production of TNF-α and IL-10 are reflective of the pro-inflammatory and anti-inflammatory functions of M1 and M2 macrophages, respectively. Their production by M1 and M2 macrophages was quantified by ELISA and summarized in Figures 5D,E. As expected, M1 macrophages produced TNF-α and M2 macrophages secreted IL-10 in healthy control individuals. However, there were no obvious differences in TNF-α and IL-10 secretion by M1 and M2 macrophages in patients with *Brucella* infection, and their respective levels were both lower than those in healthy individuals (Figure 5D). However, 3-MA treatment restored TNF-α production by M1 macrophages and IL-10 secretion by M2 macrophages in brucellosis patients (Figure 5E).

Taken together, these results indicate that the potential of monocytes to polarize into M1 or M2 macrophages is impaired in brucellosis patients, and it was recovered by 3-MA treatment.

### The Impairment of Macrophage Polarization in Patients with Brucellosis Is Associated with *Brucella*-Induced Autophagy

**Figure 6:**
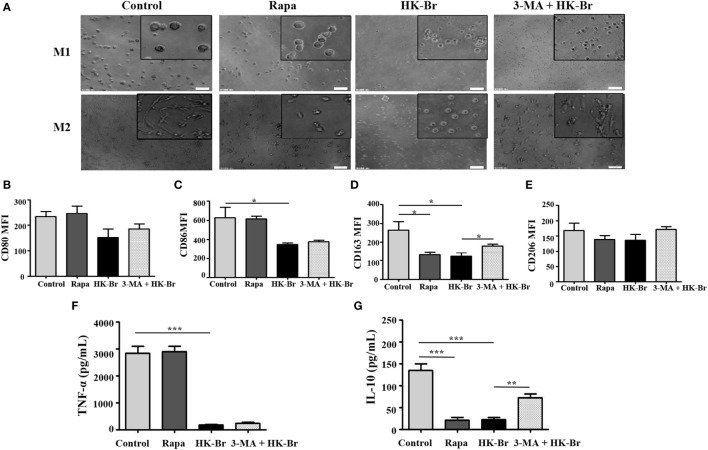
*Brucella* infection impaired the polarization of monocytes to M1 and M2 macrophages *in vitro*. Pure monocytes from five healthy volunteers were pretreated with 100 nM rapamycin or heat-killed *Brucella* (HK-Br) (MOI = 100:1) for 24 h in presence or absence of 3-MA-pretreatment, and then monocytes were differentiated to M1 or M2 macrophages. **(A)** Their morphology was observed by microscopy (scale bar 20 µm). **(B)** CD80 and **(C)** CD86 expression of M1 macrophages, **(D)** CD163 and **(E)** CD206 expression of M2 macrophages were determined by flow cytometry. **(F)** TNF-α production of M1 macrophages, **(G)** IL-10 production of M2 macrophages in the supernatants were examined by enzyme-linked immunosorbent assay. *P < 0.05, **P < 0.01, ***P < 0.001.

To determine whether *Brucella*-induced autophagy of monocytes plays a role in macrophage polarization, purified monocytes from HC were pretreated for 24 h with HK-Br. Rapamycin and 3-MA were also used as a positive control for autophagy, and an autophagy inhibitor, respectively. The pretreated monocytes were then polarized to M1/M2 macrophages as described above. M1 macrophages polarized from rapamycin-pretreated monocytes had a round-shaped appearance similar to those that received no pretreatment, while M2 macrophages from rapamycin-pretreated monocytes exhibited a smaller spindle-like shape. Interestingly, both M1 and M2 macrophages polarized from HK-Br-pretreated monocytes exhibited a smaller round shape. However, 3-MA treatment restored the morphology of M2 macrophage, but not M1 macrophage (Figure 6A). The expression levels of CD80 and CD86 on M1 macrophages (Figures 6B,C) and TNF-α production (Figure 6F) from M1 macrophages polarized from rapamycin-pretreated monocytes were similar to the controls; however, CD163 expression and IL-10 production of M2 macrophages polarized from rapamycin-pretreated monocytes were much lower than those with no pretreatment. CD86 expression on M1 macrophages and CD163 expression on M2 macrophages polarized from HK-Br-pretreated monocytes were significantly lower than those that received no pretreatment. Additionally, 3-MA treatment partially relieves the HK-Br-mediated downregulation of CD163 expression (Figure 6D) on M2 macrophages, but not CD206 expression (Figure 6E). TNF-α production by M1 macrophages and IL-10 production by M2 macrophages polarized from HK-Br-pretreated monocytes were much lower than those without pretreatment. The HK-Br-mediated inhibition of IL-10 production by M2 macrophages was recovered with 3-MA treatment (Figure 6G). Collectively, these results suggest that *Brucella*-induced autophagy affected the polarization of M2 macrophages, but not M1 macrophages.

## Discussion

Three subsets of monocytes have been identified with distinct phenotype: CD14^++^CD16^−^ subset (classical subset), CD14^+^CD16^+^ subset (non-classical subset), and CD14^++^CD16^+^ subset (intermediate subset) (32). Changes in the proportions of these monocyte subsets in blood have been observed in many disorders (33). In the present study, we found that the proportion of CD14^++^CD16^−^ monocytes in patients with brucellosis was significantly higher than in HC, whereas the proportion of CD14^+^CD16^+^ monocytes was significantly lower. In agreement with our findings, it has been reported that the proportion of CD14^++^CD16^+^ monocytes increases markedly in acute inflammation, and it could be regarded as a predictive marker for infection (34). CD14^++^CD16^+^ monocytes show a higher phagocytosis rate and secrete higher levels of IL-1β and TNF-α compared with the classical monocyte subset (CD14^++^CD16^−^) (35). However, the proportion of CD16^+^ intermediate monocytes decreases in some chronic inflammatory diseases, and the non-classical subset increases when infections have been established (36). We also found that the expression of TLR-2 (CD282) and TLR-4 (CD284) on all three monocyte subsets, particularly on the CD14^++^CD16^−^ monocyte subset, increased in brucellosis patients compared with HC. TLR-2 was reported to be activated by the L-outer membrane protein (OMP)16 and L-OMP19 of *Brucella*, and TLR-4 was activated by LPS and U-OMP16 (6). These results suggest that the expansion of the CD14^++^CD16^−^ monocyte subset is specific to *Brucella* infection; *Brucella* may manipulate monocytes *via* TLRs.

Autophagy is a cellular degradation process that captures and eliminates intracellular proteins and aged organelles by delivering them to lysosomes. This process helps cells to maintain a metabolic balance (37). We found that the expression level of an autophagy marker LC3B on monocytes in patients with brucellosis was higher than in HC, and its level decreased after 6 weeks of treatment with rifampicin and doxycycline, which are recommended as the first-line antibiotics for human brucellosis by the World Health Organization. These results indicate that *Brucella* infection induces the upregulation of LC3B on CD14^++^CD16^+^ monocyte subset. Furthermore, we found that *Brucella* infection inhibited the production of pro-inflammatory and anti-inflammatory cytokines by monocytes stimulated by LPS, and such inhibition was relieved by an inhibitor of LC3B-related autophagy, 3-MA. Therefore, our data confirm that *Brucella* induce LC3-related autophagy. Recent studies showed that autophagy plays a modulatory role in microbial infection. Some pathogens induce complete autophagic response and then died from it, whereas some other pathogens draw benefit from an incomplete autophagic response, which enable their survival and proliferation (30). Previous *in vitro* studies have demonstrated that autophagy facilitates *Brucella* survival (38), through unknown mechanisms. LC3 has been regarded as a major marker of autophagy; however, the role of LC3-related autophagy in *Brucella* infection remains controversial. Some studies reported that LC3 does not participate in the autophagy induced by *Brucella* infection (9). but other studies demonstrated that the *Brucella*-induced autophagic response is LC3-related (38).

Circulating monocytes can differentiate into macrophages, which can be classified into classically activated macrophages (M1) and alternatively activated macrophages (M2) (39). M1 macrophages promote type 1 immune responses through the synthesis of pro-inflammatory cytokines and chemokines, such as TNF-α, IL-1β, IL-12, IL-18, CCL15, CCL20, CXCL8-11, and CXCL13 (40). M2 macrophages participate in type 2 immune responses (41). M1/M2 polarization of macrophages in inflammation and its resolution has been described for some diseases (19), but not for *Brucella* infection. We found that the potential of monocytes to polarize into M1 or M2 macrophages was impaired in brucellosis patients, and the impairments were reversed by inhibiting LC3B-related autophagy. To our knowledge, this is the first description of macrophage polarization in brucellosis patients. These results suggest that *Brucella* infection suppresses monocyte differentiation to both M1 and M2 macrophages by inducing LC3B-related autophagy.

Our results are in agreement with other studies reporting that autophagy influences the polarization of macrophages and down-regulates inflammation. Defects in macrophage autophagy may promote inflammatory disease. For instance, a decrease in macrophage autophagy in obesity leads to hepatic inflammation and the progression of liver injury (42), and specific proteins could induce autophagy and facilitate M2-type polarization of tumor-associated macrophages (43). We found that HK-Br and rapamycin upregulate LC3B expression on monocytes. Rapamycin only suppresses monocyte polarization to M2 macrophages, and HK-Br inhibits monocyte polarization to both M1 and M2 macrophages. Rapamycin is a macrocyclic triene antibiotic, now considered an immunosuppressive agent for the prevention of kidney transplant rejection (44), and an autophagy inducer that is often used as a positive control for autophagic response. Rapamycin can induce LC3-dependent autophagy in macrophages and epithelial cells (45). Recently, it was also shown that rapamycin affects M2 survival and diminishes the M1-like inflammatory responses in patients with type I diabetes (46). Therefore, our results are consistent with the idea that *Brucella* inhibits M2 macrophage polarization by inducing LC3B-related autophagy. The specific mechanisms by which *Brucella* infection inhibits M1 macrophage polarization remain unknown.

In summary, we found that *Brucella* infection led to an increase in the proportion of CD14^++^CD16^−^ monocytes, a decrease in the proportion of CD14^+^CD16^+^ monocytes, and increased expression of the autophagy-related protein LC3B in CD14^++^CD16^−^ monocytes obtained from brucellosis patients. In addition, *Brucella* infection inhibited the production of pro-inflammatory and anti-inflammatory cytokines by monocytes *via* LC3-dependent autophagy. Moreover, *Brucella* infection inhibited macrophage polarization, and 3-MA, an inhibitor of LC3B-related autophagy, partially restored macrophage polarization in patients with brucellosis. Intriguingly, we also found that the upregulation of LC3B expression by rapamycin and HK-Br *in vitro* inhibited M2 macrophage polarization, and 3-MA partially restored the macrophage polarization. Taken together, these findings reveal that *Brucella* dysregulates monocyte and macrophage polarization through LC3B-dependent autophagy. Immune modulation through this pathway may lead to the development of new therapeutics. Brucellosis is caused by several species from *Brucella* genre, but we have no evidence to conclude that the findings in this study extends for different *Brucella* species, a relation between the bacterial pathogenicity and its effect on LC3-induced autophagy needs to be further explored as well in the future.

## Ethics Statement

The study was approved by the Human Research Ethics Committee of the First Hospital of Jilin University, Jilin Province, China. All adult subjects provided written informed consent, and no children were included in the study.

## Author Contributions

YW, YL, and HL: planning and performing the experiments, analysis data, writing the paper; HS: performing the qRT-PCR experiment; NZ: performing the FACS experiment; LL, FW, and KZ: collection of clinical materials; WB, XJ, and LS: interpretation and editing; ZT: experimental design, interpretation, funding, and writing.

## Conflict of Interest Statement

The authors declare that the research was conducted in the absence of any commercial or financial relationships that could be construed as a potential conflict of interest.
