# Supplementary figures and images for: Brucella Dysregulates Monocytes and Inhibits Macrophage Polarization through LC3-Dependent Autophagy
Source: Front Immunol. 2017 Jun 12;8:691. doi: 10.3389/fimmu.2017.00691 (PMC5467008; doi:10.3389/fimmu.2017.00691)

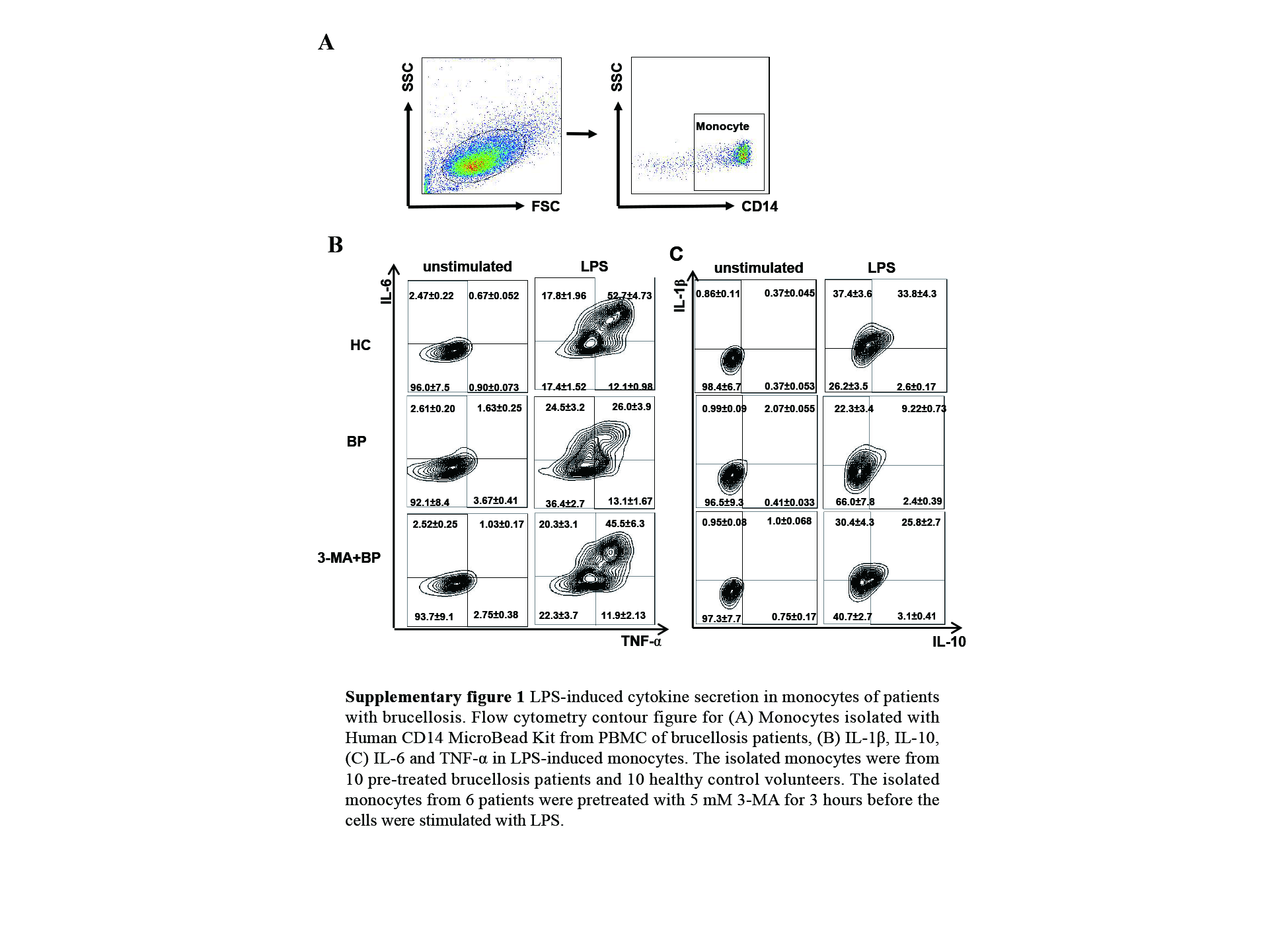

Supplement: Supplementary file 1 [file Image_1.TIF]

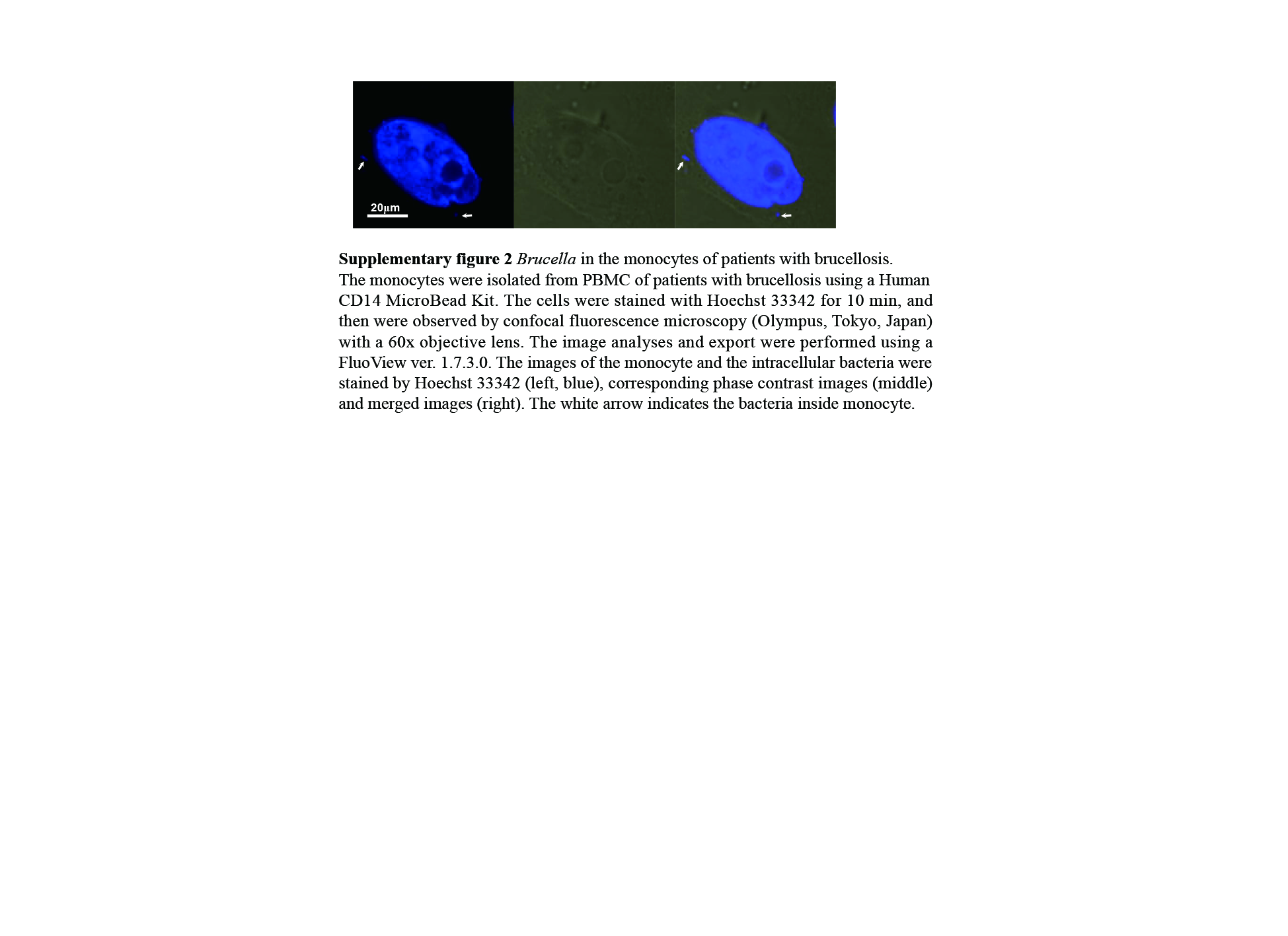

Supplement: Supplementary file 2 [file Image_2.TIF]

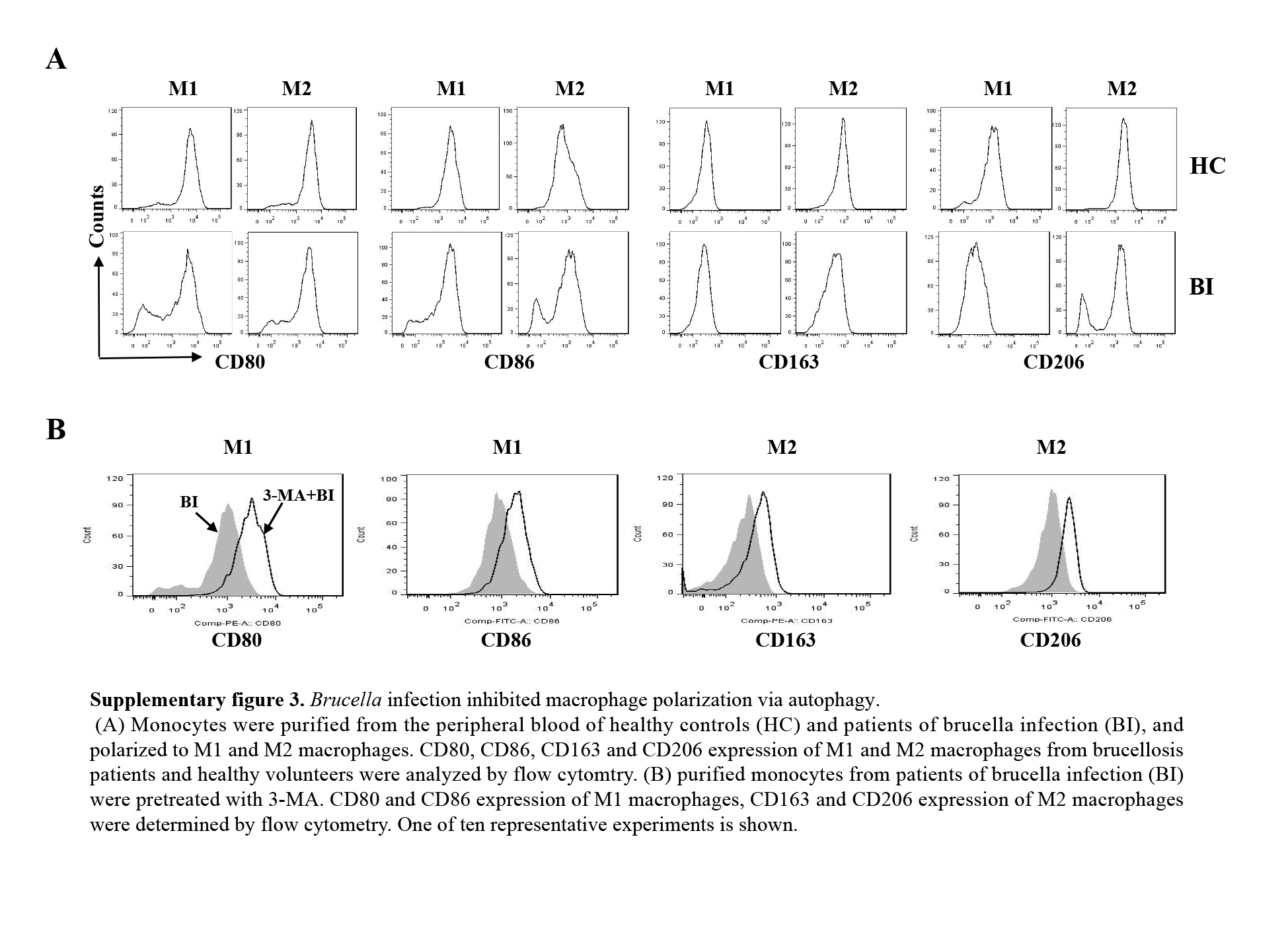

Supplement: Supplementary file 3 [file Image_3.TIF]

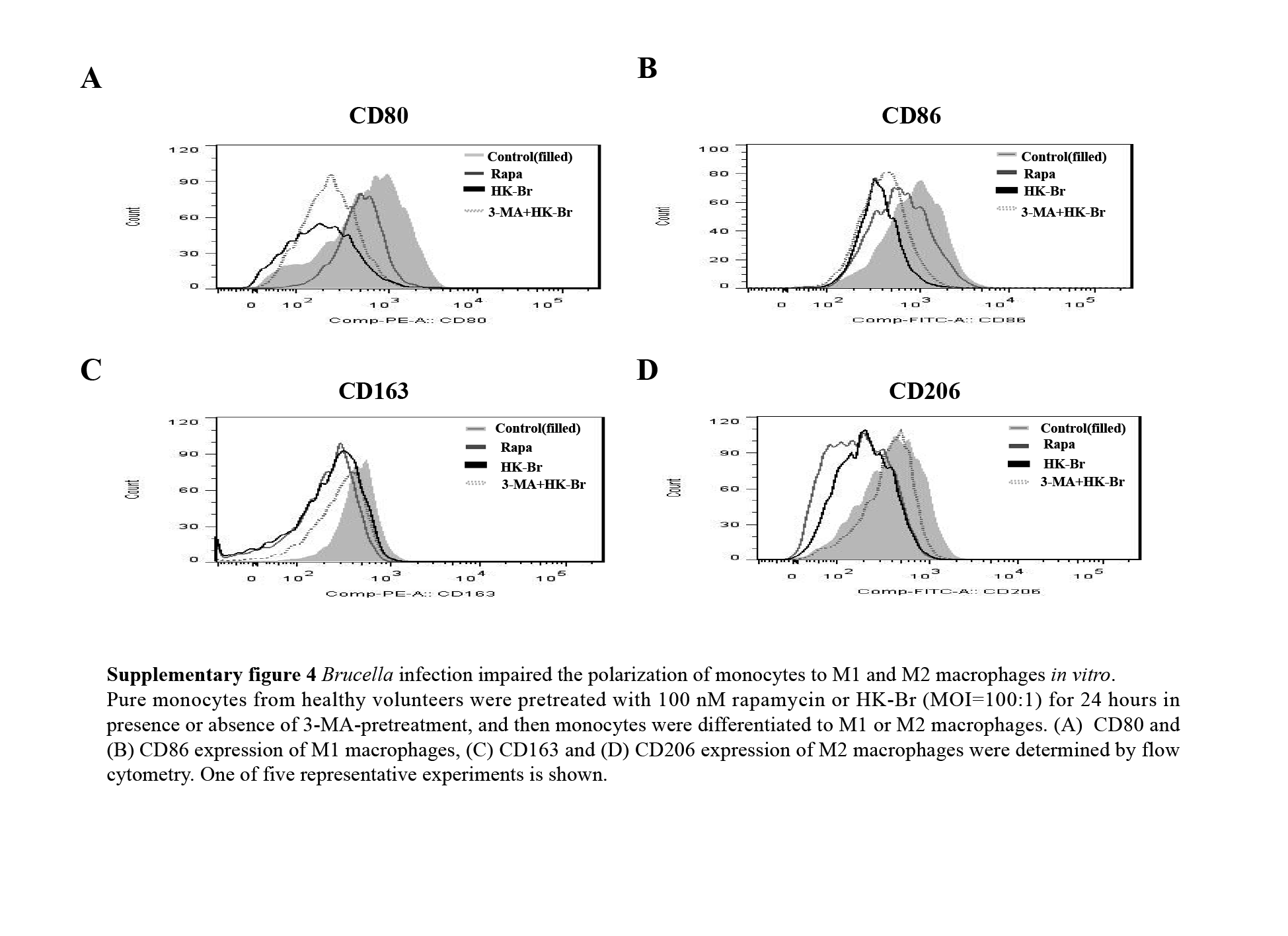

Supplement: Supplementary file 4 [file Image_4.TIF]
